# Can You See Us Play? Observing Inclusive Outdoor Play Behaviour Among Children With and Without Disabilities: A Mixed Methods Study

**DOI:** 10.1111/cch.70198

**Published:** 2025-12-04

**Authors:** R. Q. Beekhuizen, M. A. T. Bloemen, E. A. M. Bolster, K. Visser, R. van der Lugt, M. Bassa, N. L. Henry, E. M. W. Kotte, M. van Hartingsveldt, J. W. Gorter

**Affiliations:** ^1^ Research Group Moving, Growing and Thriving Together University of Applied Sciences Utrecht Utrecht the Netherlands; ^2^ Institute of Human Movement Studies, Master Paediatric Physiotherapy University of Applied Sciences Utrecht Utrecht the Netherlands; ^3^ Department of Human Geography and Spatial Planning, Faculty of Geosciences Utrecht University Utrecht the Netherlands; ^4^ Research Group Co‐Design University of Applied Sciences Utrecht Utrecht the Netherlands; ^5^ Marry Bassa Graphic and Product Design Beesd the Netherlands; ^6^ De Hoogstraat Rehabilitation Utrecht the Netherlands; ^7^ Fitkids Foundation Amsterdam the Netherlands; ^8^ Department of Occupational Therapy, Faculty Health, Sports and Physical Activity Amsterdam University of Applied Sciences Amsterdam the Netherlands; ^9^ Department of Rehabilitation, Physical Therapy Science and Sports, UMC Utrecht Brain Center University Medical Center Utrecht Utrecht the Netherlands

**Keywords:** children, disability, inclusion, observation, outdoor play, Test of Environmental Supportiveness, Test of Playfulness

## Abstract

**Background:**

Outdoor play is essential for children's development. However, children with disabilities face barriers in outdoor play. In the Netherlands, there are limited opportunities for children with and without disabilities to play together. While previous research examined the perspectives of parents, professionals and children regarding inclusive outdoor play, little is known about actual outdoor play behaviour. This exploratory study uses observational research to investigate how children with and without disabilities engage in outdoor play together.

**Methods:**

We used a mixed‐methods approach: quantitative assessments using the Test of Playfulness (ToP) and the Test of Environmental Supportiveness (TOES) were combined with qualitative observations focused on inclusive outdoor play, conducted during six outdoor play sessions at inclusive playgrounds. Children with and without disabilities aged 4–12 years (*n* = 63) were selected through purposive sampling.

**Results:**

Quantitative analysis revealed that boys and children with disabilities scored significantly lower on the ToP, indicating reduced playfulness. Gender was also significantly associated with TOES scores, with boys scoring lower, indicating less environmental support for play. Qualitative observations identified six key themes influencing inclusivity in outdoor play behaviour: (1) variety of play types, (2) getting to know each other, (3) making contact, (4) interaction while playing together, (5) influence of the social environment and (6) influence of the physical environment.

**Conclusions:**

This study highlights the diversity in playfulness skills and environmental support observed among children with and without disabilities. It emphasizes the role of social interactions, peer relationships and environmental factors in shaping inclusive play behaviour. These findings underscore the importance of both social and physical aspects to promote inclusive outdoor play. By integrating these insights, the study provides guidance for paediatric rehabilitation professionals, whose role in facilitating inclusive play and overcoming barriers is essential to create outdoor play opportunities for all children.

## Summary

- Children with disabilities engage in a wide range of outdoor play activities but face more physical and social barriers that limit inclusive participation.
- Gender and disability status significantly affect playfulness, while gender also significantly influences environmental supportiveness during outdoor play.
- Limited social inclusion during play can result from unfamiliarity rather than rejection. Children tend to play with peers they already know, regardless of disability status.
- Inclusive outdoor play requires more than physical accessibility, and it demands social support, peer understanding and acceptance and professional guidance.

## 1 Introduction

Children worldwide are spending less and less time outdoors (Lee et al. 2021). In the Netherlands, more than half of all children wish they could play outside more often, and only 13% plays outside daily (Verian 2024). This decline is concerning because outdoor play positively impacts children's physical, psychosocial and cognitive development, as well as their overall well‐being and quality of life (de Vries 2012; Gill 2014; Martin et al. 2022; Tremblay et al. 2015). Play is also valuable for its own sake: It brings joy and freedom. While adults often emphasize the developmental and educational aspects of play, children themselves value the autonomy, excitement, choices and opportunities for social interaction that play affords (Buldu and Buldu 2023; Morgenthaler et al. 2023; Prellwitz and Skär 2007).

Although the decline in outdoor play affects all children, not all have equal opportunities to play outside; children with disabilities in particular often face barriers to outdoor play (Atkinson et al. 2017; Engelen et al. 2021; Schreuer et al. 2013). This is concerning given that every child has the right to play, as outlined by the United Nations Convention on the Rights of the Child (Bianguin 2018; Convention on the Rights of the Child 1989). Children with disabilities face physical and social barriers that limit their participation in outdoor play activities (Barron et al. 2017; Engelen et al. 2021; Morgenthaler et al. 2023; Visser et al. 2025). Physical barriers include environments that are not accessible or insufficiently challenging. Social barriers relate to difficulties in establishing interactions between children with and without disabilities, which may be influenced by disability‐based stigma, including negative feelings and expectations regarding the physical and social abilities of children with disabilities (Beekhuizen et al. 2025; Bloemen et al. 2015; Engelen et al. 2021; Hanes et al. 2019; Morgenthaler et al. 2023).

While this stigma is recognized internationally (Gorter et al. 2014; Morgenthaler et al. 2023; Soper et al. 2025; Vignes et al. 2009), the Dutch educational system, in which many children with disabilities attend regional special primary education (SPE) instead of local regular primary education (RPE), reduces opportunities for both formal and informal interactions, limiting their access to developmentally supportive social experiences (Beekhuizen et al. 2025; Engelen et al. 2021; Verschuren et al. 2012; Visser et al. 2025). This makes the current situation in the Netherlands a valuable setting to study inclusive outdoor play and understand how children with and without disabilities interact in practice. In this paper, we focus specifically on children's participation in outdoor play as one important dimension of inclusion, recognizing that inclusion more broadly entails belonging, acceptance and equitable access to opportunities that support developmentally meaningful engagement (Babik and Gardner 2021; Gorter et al. 2014; Macmillan et al. 2013).

Paediatric rehabilitation professionals, such as paediatric physical therapists (PPTs) and occupational therapists (OTs), can play a crucial role in facilitating inclusive outdoor play by, for example, fostering early inclusive experiences and encouraging interactions between children with and without disabilities (Engelen et al. 2021; Reedman et al. 2017). However, they often face challenges due to a lack of knowledge of how to effectively support inclusive outdoor play in daily practice (Engelen et al. 2021; Visser et al. 2025). To tailor interventions, rehabilitation professionals need understanding of how both the physical and social environments impact play opportunities (Loebach and Cox 2020).

To advance this understanding, previous studies have explored the perspectives of children with and without disabilities, parents and health and welfare professionals regarding inclusive outdoor play (Beekhuizen et al. 2025; Engelen et al. 2021; Longo et al. 2020; Morgenthaler et al. 2023; Visser et al. 2025). These studies aimed to identify facilitators and barriers to inclusive outdoor play through interviews. Yet, there may be discrepancies between what children report about their experiences and expectations and how they actually play when interacting with each other (Beekhuizen et al. 2025; Hestenes and Carroll 2000). To date, no studies have observed inclusive outdoor play behaviour among children in the Netherlands.

Conducting observational research could provide valuable additional insights into factors that facilitate or hinder inclusive outdoor play behaviour (Beekhuizen et al. 2025; Bundy et al. 2001; Cacciattolo 2015). This offers potential directions for interventions and enables paediatric rehabilitation professionals to support children in participating in inclusive outdoor play. The research question posed in this study is: ‘How do children aged 4 to 12, with and without disabilities, engage in inclusive outdoor play?’ Additionally, it investigates associations between inclusive outdoor play and potential influencing factors such as age, gender, school setting and other relevant variables. Based on previous literature, we expected that both children's playfulness and environmental supportiveness in inclusive outdoor play would be influenced by physical and social facilitators and barriers (Beekhuizen et al. 2025; Engelen et al. 2021).

## 2 Methods

### 2.1 Research Procedure

In this exploratory study we used a mixed‐methods approach. We quantitatively assessed children's playfulness and environmental factors that influence their ability to play (part I), and we conducted qualitative observations of the inclusive outdoor play behaviour (part II). We applied the criteria from Strengthening the Reporting of Observational Studies in Epidemiology (STROBE) and the Standards for Reporting Qualitative Research (SRQR) to enhance transferability and transparency (Elm et al. 2007; O'Brien et al. 2014).

#### 2.1.1 Part I—Quantitative Assessment

Because participation in inclusive outdoor play is influenced by multiple factors, we examined associations between children's playfulness and environmental play scores and several potential influencing factors, including age, gender, play location, presence of a disability and use of assistive devices. The aim was to identify which of these factors are most strongly associated with variations in children's playfulness, as measured by the Test of Playfulness version 4.0 (ToP), and in environmental supportiveness, as measured by the Test of Environmental Supportiveness (TOES) (Bundy et al. 2001, 2009; Spaargaren 2011).

#### 2.1.2 Part II—Qualitative Observation

Qualitative observations were integrated to provide deeper insight into children's inclusive outdoor play behaviour and to support the interpretation of quantitative findings by contextualizing how specific factors manifest in actual play situations.

### 2.2 Setting

**FIGURE 1:**
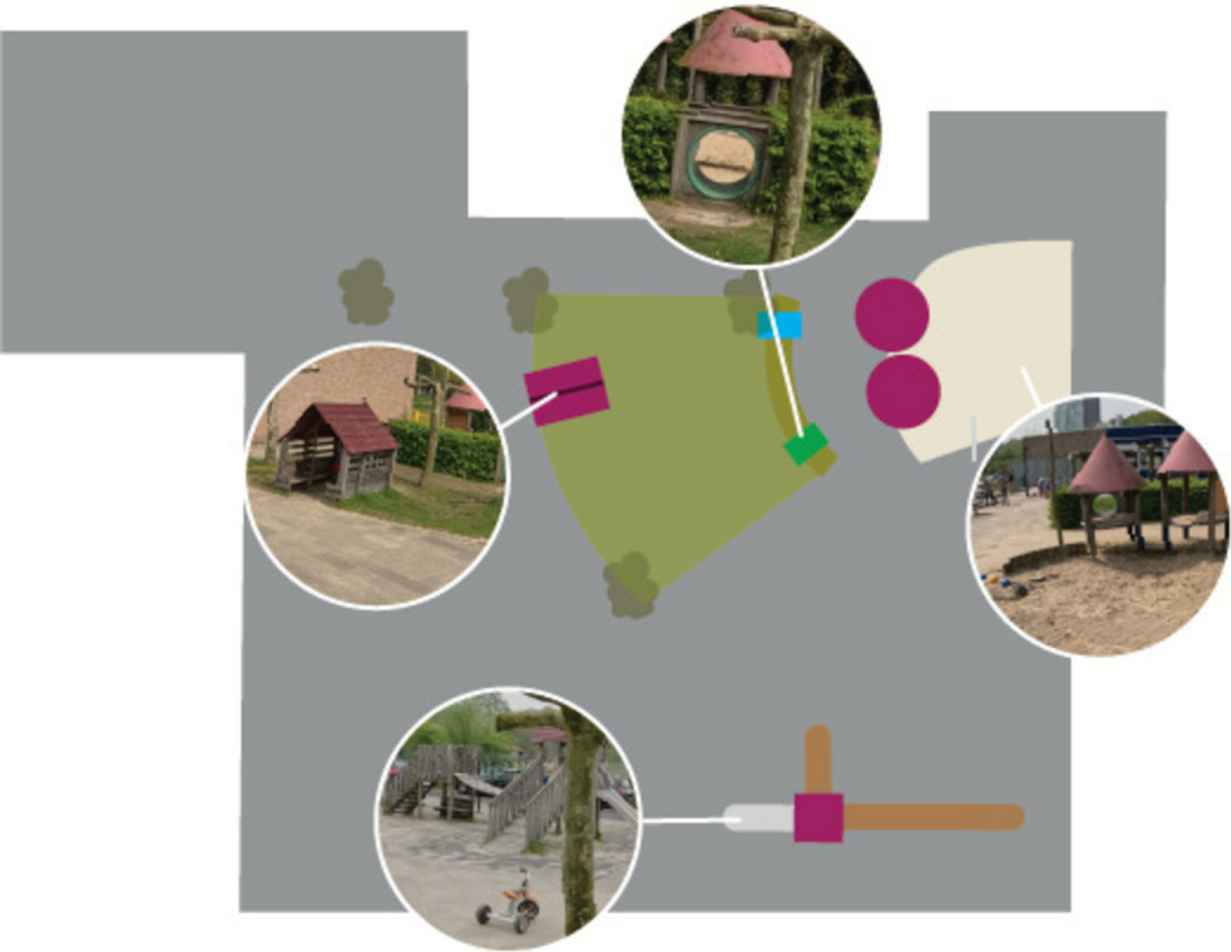
Design playground Utrecht lower grade.

**FIGURE 2:**
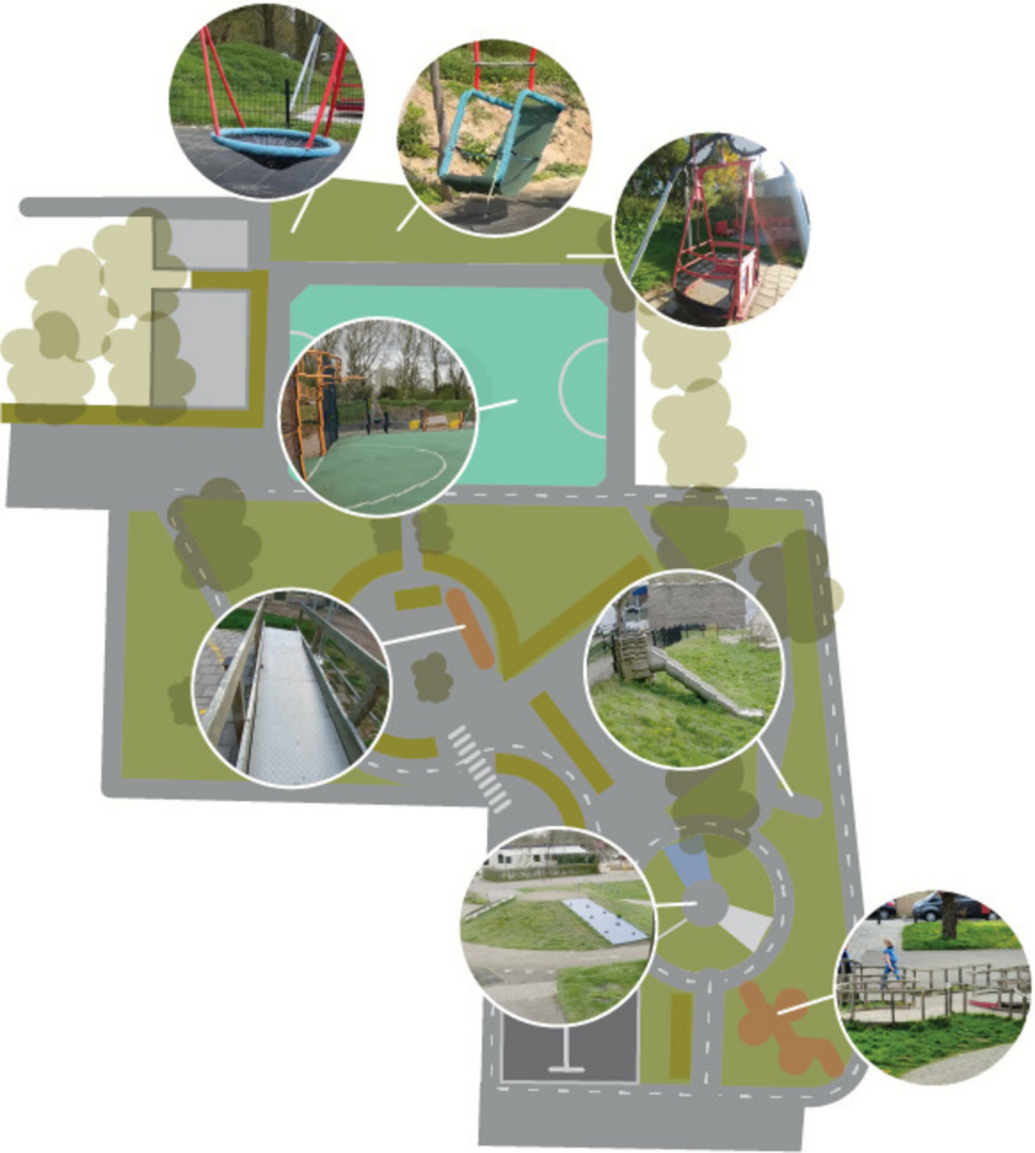
Design playground Utrecht middle and upper grade.

**FIGURE 3:**
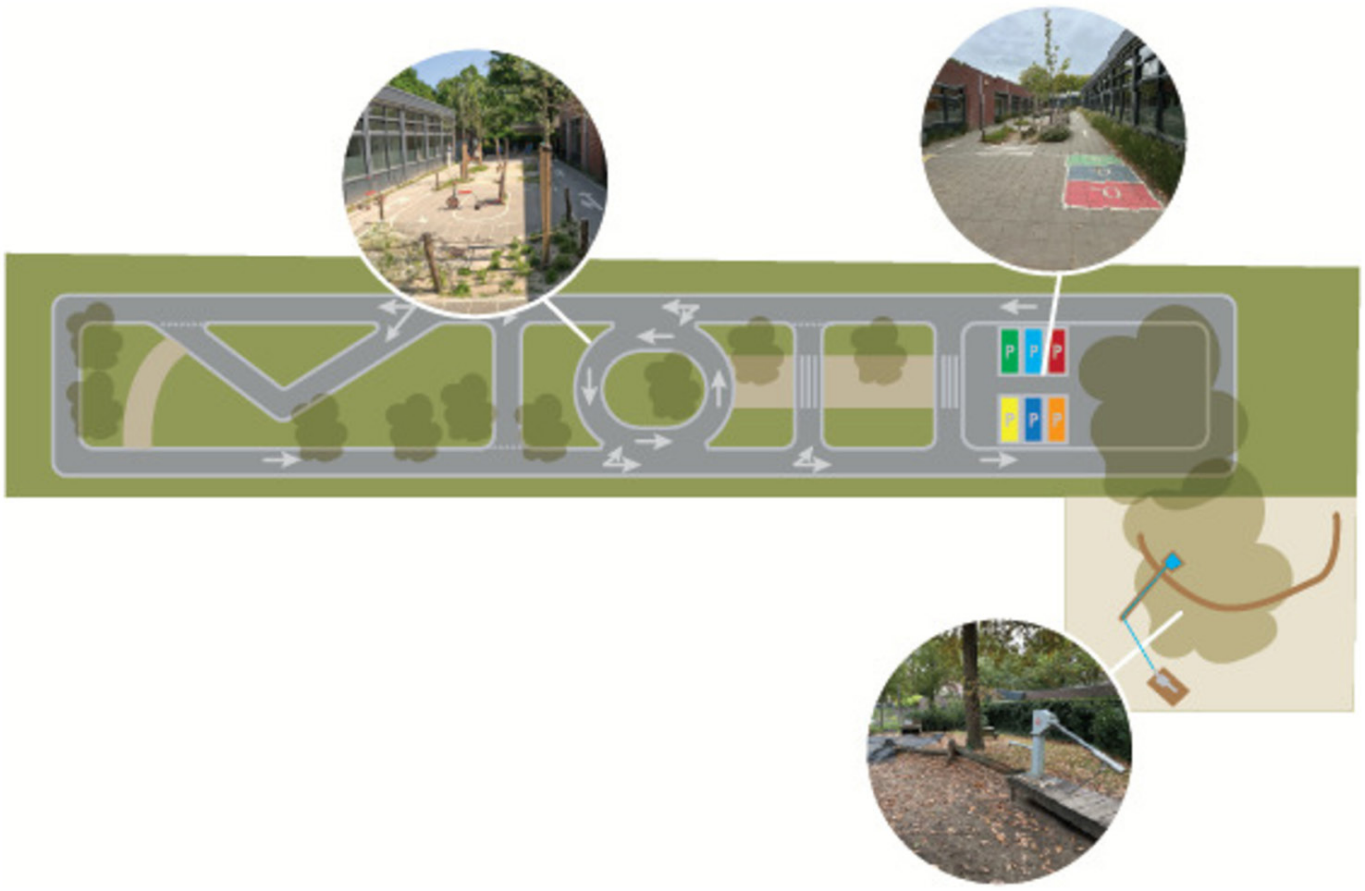
Design playground Breda lower grade.

**FIGURE 4:**
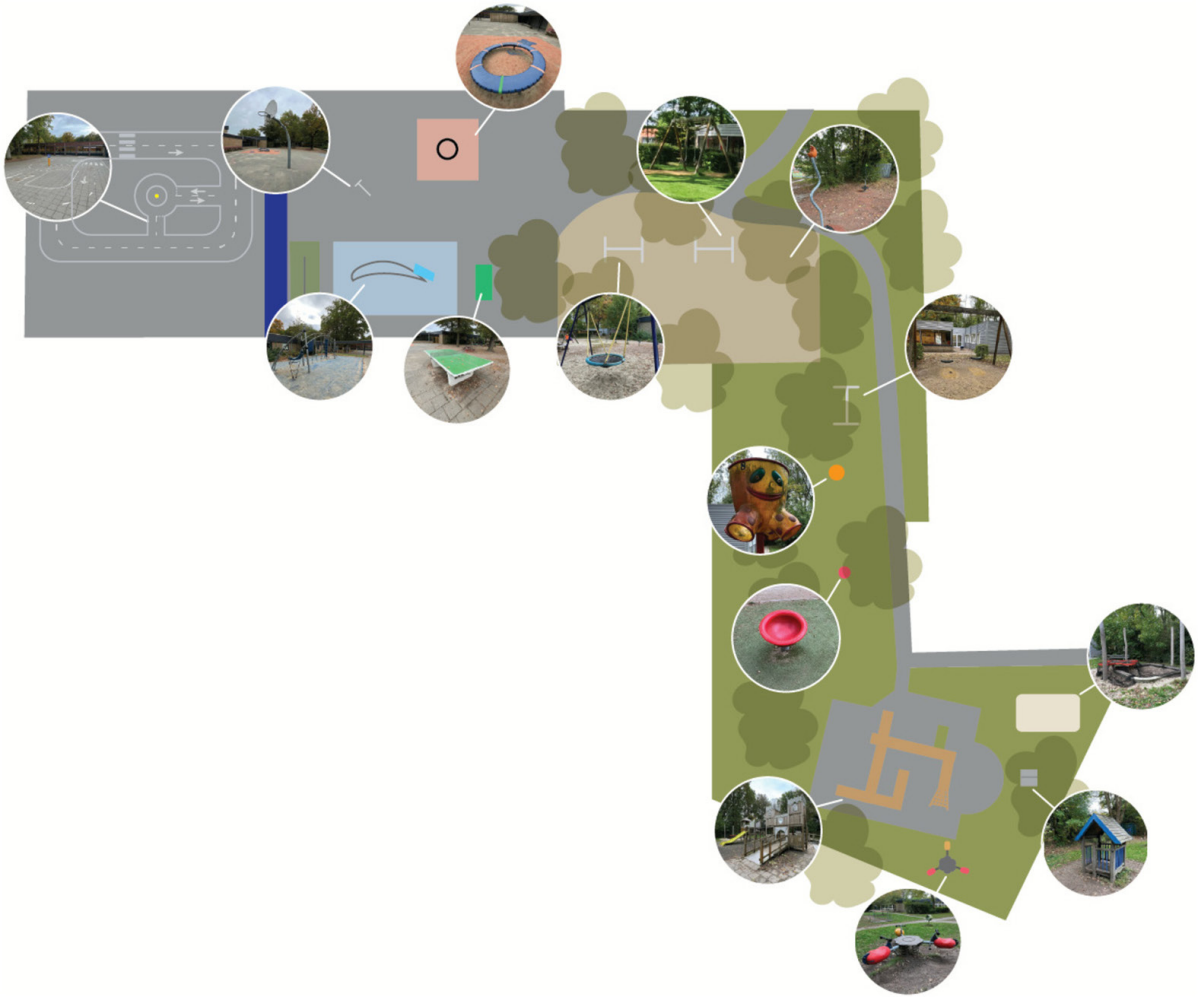
Design playground Breda middle and upper grade.

We conducted data collection during six scheduled outdoor play sessions. All sessions were held on weekdays during April–June 2023, in mild (12°C–20°C) and dry spring weather, allowing unrestricted outdoor play. Sessions were planned to ensure the participation of children with disabilities. Children were assigned to one play session according to three age categories: lower (kindergarten), middle (grades 1–3) and upper (grades 4–6). The sessions took place on playgrounds of two SPE schools in two cities in the central and southern Netherlands. Three sessions were held at each location. These playgrounds were selected for their accessibility and inclusive design. Sessions included age‐appropriate environments and inclusive materials (e.g., balls, go‐karts and bikes) (see Figures 1, 2, 3, 4). Most children with disabilities were familiar with the playgrounds, while most children without disabilities were visiting them for the first time. Children had the freedom to choose their own play activities and use the entire playground. We aimed for groups of approximately 10 children per session, with an equal distribution of children with and without disabilities. Familiar teachers and an orthopedagogue of SPE were present to supervise and to ensure a safe environment. Parents were present during the sessions for children in lower grades but refrained from interfering in the play to avoid disrupting the children.

### 2.3 Participants

Participants (*N* = 63) were Dutch children of primary school age, ranging from 4 to 12 years old (*M* = 8.00, SD = 2.48). Using purposive sampling, we ensured heterogeneity in age, gender, residence, school setting, presence and type of disability and use of assistive devices (Boeije and Blijenbergh 2019; Hodkinson 2007, 2011; Kalyva and Agaliotis 2009). Recruitment occurred via flyers distributed in schools, professional networks of PPTs and researchers, and through LinkedIn. Of the 63 participating children, 60 knew one or more other participants prior to the session, whereas three did not know any of the other children. To facilitate social interaction, children were allowed to invite a friend to participate in the session.

### 2.4 Ethics

The Research Ethics Screening Committee in Health at the University of Applied Sciences Utrecht advised that this study was exempt from the Medical Research Involving Human Subjects Act (file number: 221‐000‐2022). All participants and their parents received written information, and parental informed consent was obtained, as all children were under the age of 12. To protect anonymity, children wore numbered or coloured vests that did not reveal disability status. Some physical disabilities were visible during the sessions, but others (e.g., autism) were not and could only be identified in the dataset afterward.

### 2.5 Data Collection and Measurement

#### 2.5.1 Part I—Quantitative Assessment

The ToP is an observational assessment that evaluates a child's playfulness based on five elements: intrinsic motivation, internal control, shared control, freedom to suspend reality and framing. It consists of 21 items rated on a 4‐point scale (Skard and Bundy 2008; Spaargaren 2011). The TOES measures how supportive the physical and social environment is for a child's play and consists of 17 items, also rated on a 4‐point scale (Bundy et al. 2009; Spaargaren 2011). Both tools are used in paediatric research and practice, and their reliability and validity have been well established (Bronson and Bundy 2001; Bundy et al. 2001, 2009; Skard and Bundy 2008).

During the six outdoor play sessions, nine trained observers independently assessed up to four children at a time using the ToP and TOES. The observers included researchers and students with backgrounds in PPT, orthopedagogy, co‐design and human geography. They were trained by researchers involved in the Dutch adaptation of the ToP and TOES, with joint scoring exercises during the course to promote inter‐rater consistency. The lead researcher and PPT (RB), who also works in practice with children with and without disabilities, reviewed video recordings of the play sessions to check all ratings. Any discrepancies were resolved through discussion with the original observer, or, if needed, in consultation with a third PPT researcher (MB). Raw scores were calculated for each child and used for the statistical analyses.

#### 2.5.2 Part II—Qualitative Observation

To provide deeper insight into children's inclusive outdoor play behaviour, we developed a thematic observation form based on existing literature on inclusive outdoor play (Babik and Gardner 2021; Engelen et al. 2021; James et al. 2022). Since the ToP and TOES do not specifically focus on interactions between children with and without disabilities, the observation form included key themes such as playing together, interactions, physical environment and types of play (see Appendix S1).

Observers were stationed at fixed playground zones and recorded their field notes verbally using Philips Voice Tracer DVT1110 recorders. These observers were distinct from the raters who scored ToP and TOES.

### 2.6 Data Management and Analysis

#### 2.6.1 Part I—Quantitative Assessment

We divided the dataset into two subsets to analyse the response variables separately: raw ToP score (TOPRS) and raw TOES score (TOESRS). Each subset included the same independent variables: play location, gender, age category (Agecat), presence of a disability and use of assistive devices (Aidcat).

1. Preliminary Correlation Analysis
  We computed Pearson correlation matrices to explore linear relationships among the independent variables. Heatmaps were generated to visualize correlations and assess multicollinearity.
2. Linear Regression Analysis
  Separate multiple linear regression models were fitted for each response variable using the ordinary least squares (OLS) method (Zdaniuk 2014). Each model included a constant term. Variables with *p*‐values < 0.05 were considered statistically significant (Kwak 2023). For each model, 95% confidence intervals (CIs) were calculated.

#### 2.6.2 Part II—Qualitative Observation

Each observation was transcribed verbatim using Amberscript and pseudonymized. The data were independently analysed in ATLAS.ti 23 for Windows by one researcher (RB) and two PPT master's students. All researchers had received training in qualitative research methods prior to the analysis.

We followed an inductive approach, guided by the six‐step process outlined in Braun and Clarke's reflexive thematic analysis (Braun and Clarke 2019). Initially, we thoroughly read the transcripts to gain familiarity with the dataset (step 1). Subsequently, we independently identified segments and generated initial codes (step 2). Through critical peer review, consensus was reached; if no consensus was achieved, a fourth PPT researcher was consulted (MB). These codes were then grouped into potential main‐ and sub‐themes by three PPT researchers (RB, EB and MB) to ensure investigator triangulation and trustworthiness (step 3) (Korstjens and Moser 2018). Following this, we conducted a comprehensive review of the themes in a peer debriefing session with all authors, defining and naming the themes (step 4–5). Finally, we selected quotes and finalized the analysis (step 6).

## 3 Results

Overall, the inclusive outdoor play behaviour of 33 children with disabilities and 30 children without disabilities was observed across six different outdoor play sessions. Each play session lasted between 45 and 60 min. Among the 33 children with disabilities, one diagnosis was unknown, and 11 used an assistive device. The participants' characteristics are detailed in Table 1.

**TABLE 1:** Participant characteristics by outdoor play session and grade.

Grade	Outdoor play session	Location	Participants (*n*)	Age, mean (range)	Gender (B/G)	Education (RPE/SPE)	Disability (CWD/CWOD)	Types of condition	Mobility aid (yes/no)
Lower	Session 3	Utrecht	11	4.6 (4–6)	5/6	7/4	4/7	CP (*n* = 3), MT9 + CVI	3/8
	Session 5	Breda	9	5.4 (5–6)	7/2	4/5	5/4	AMC, CP, ex‐preterm DD + CFD, SLD + dyspraxia, SLD + suspected ASD	1/8
Middle	Session 2	Utrecht	11	7.6 (6–10)	4/7	5/6	6/5	CP (*n* = 4), DMD, KGB + ADHD + DD	2/9
	Session 6	Breda	10	7.3 (6–9)	2/8	4/6	6/4	ASD + MID, CHARGE, CP, PAE + ABI, TS, WS + DD	1/9
Upper	Session 1	Utrecht	12	10.7 (10–12)	7/5	7/5	7/5	CP + Ataxia, CP + CVI, DMD, diagnose unknown, PWS, SCD, UCMD	3/9
	Session 4	Breda	10	10.1 (7–11)	4/6	5/5	5/5	ASD + ADD + DCD, ASD + SLD, ASD + suspected DCD, chronically ill + cleft, CP	1/9

*Note:* Utrecht and Breda refer to locations in different regions where inclusive outdoor play sessions were conducted.

Abbreviations: ABI = acquired brain injury, ADHD = attention‐deficit/hyperactivity disorder, AMC = arthrogryposis multiplex congenita, ASD = autism spectrum disorder, B = boys, CFD = complex feeding difficulties, CHARGE = CHARGE syndrome, CP = cerebral palsy, CVI = cortical visual impairment, CWD = children with disabilities, CWOD = children without disabilities, DCD = developmental coordination disorder, DD = developmental delay, DMD = Duchenne muscular dystrophy, G = girls, KGB = KBG syndrome, MID = mild intellectual disability, MT9 = mosaic trisomy 9, *n* = number of participants, PAE = post‐anoxic encephalopathy, PWS = Prader–Willi syndrome, RPE = regular primary education, SCD = suspected chromosomal disorder, SLD = speech and language disorder, SPE = special primary education, TS = Troyer syndrome, UCMD = Ullrich congenital muscular dystrophy, WS = West syndrome.

### 3.1 Part I—Quantitative Assessment

ToP scores ranged from 16 to 69 (*M* = 48.92, SD = 13.19), within a possible range of 0–81. Higher scores on the ToP indicate more playful behaviour. TOES scores ranged from 1 to 22 (*M* = 12.63, SD = 4.59), with higher scores reflecting a more supportive play environment. The possible score range for the TOES is −34 to +34.

1 Preliminary correlation analysis

2 Linear regression analysis

**FIGURE 5:**
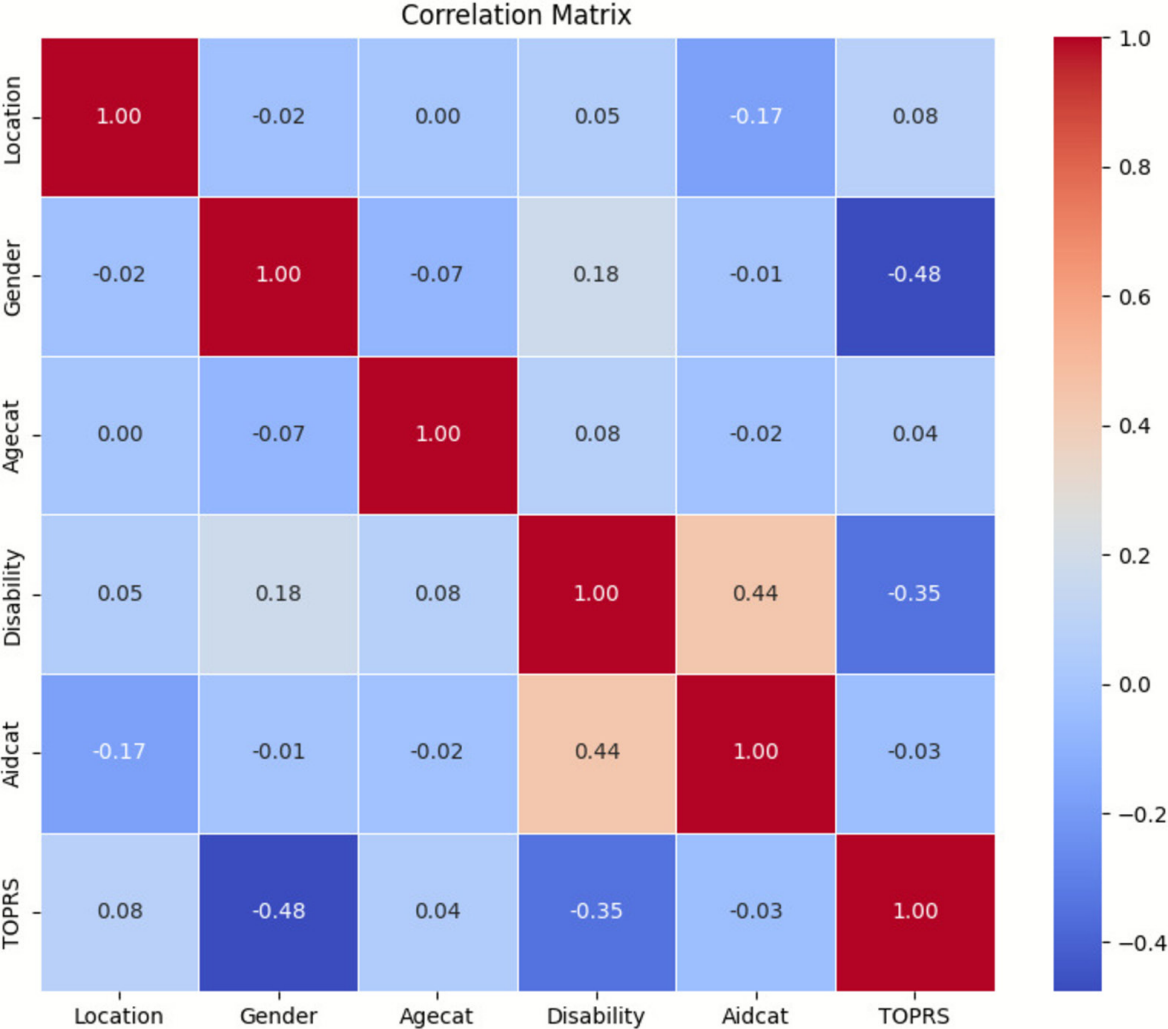
Correlation matrix ToP.

**FIGURE 6:**
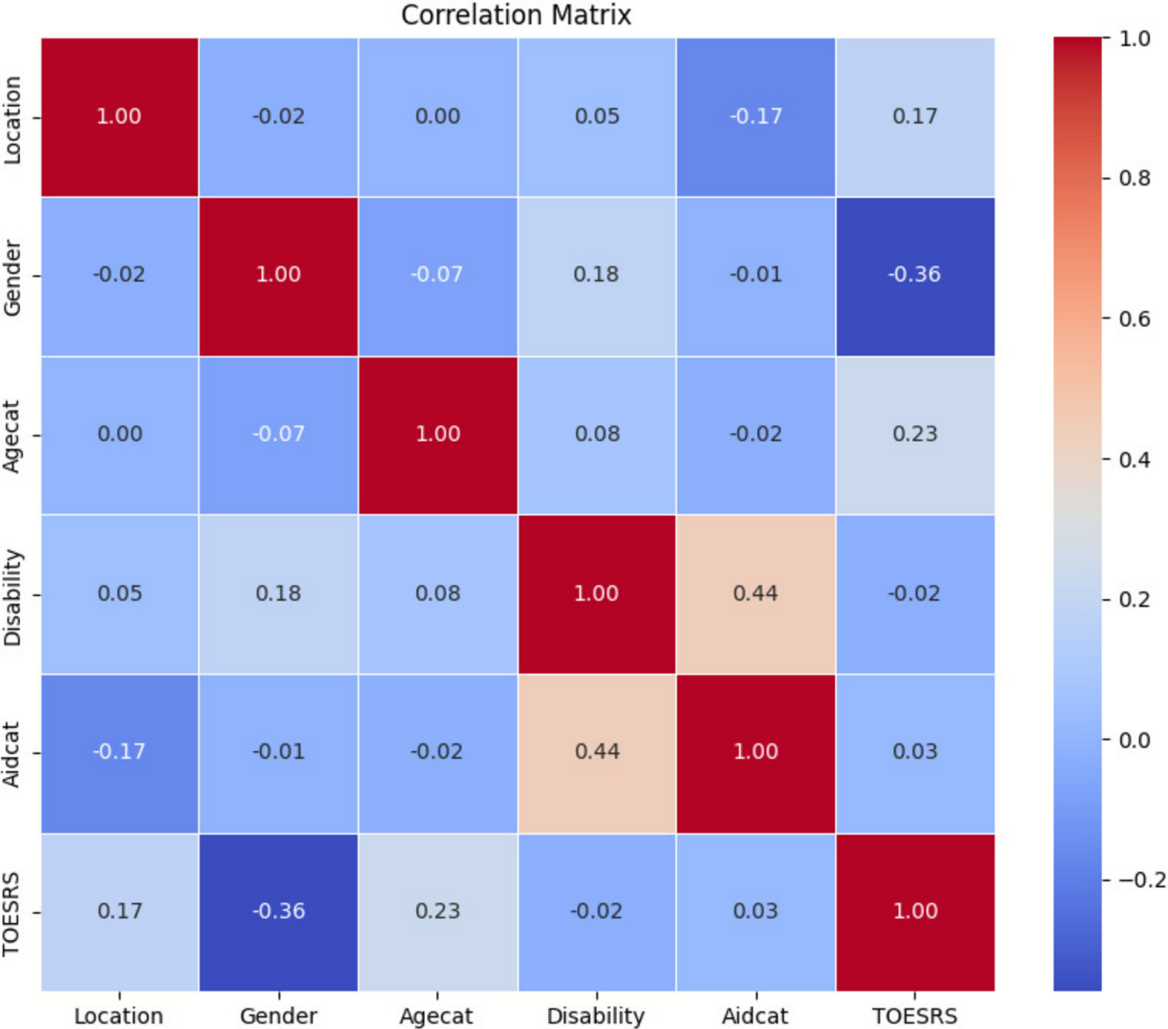
Correlation matrix TOES.

For both the ToP raw scores (TOPRS) and the TOES raw scores (TOESRS), preliminary Pearson correlation analyses indicated that the highest correlation coefficient among the independent variables was below 0.49 (see Figures 5 and 6). This suggests that none of the variables were strongly correlated, and therefore, all were retained for the regression analyses (Shrestha 2020).

Separate multiple linear regression models were fitted for each response variable using the ordinary least squares (OLS) method.

The linear regression analysis for TOPRS identified two statistically significant associated variables. First, gender was negatively associated with ToP scores (*p* = 0.001), indicating that boys scored significantly lower on playfulness than girls. Second, the presence of a disability was also significantly negatively associated (*p* = 0.009), indicating that children with disabilities had lower ToP scores compared to children without disabilities. Other variables, including play location, age category and use of an assistive device, were not significantly associated with ToP scores (all *p* > 0.05) (see Table 2).

**TABLE 2:** OLS multiple regression results ToP.

Variable	Coefficient	Std. error	*t*	*p*	95% CI lower	95% CI upper
Intercept	55.68	3.26	17.11	< 0.001	49.16	62.20
Location	2.97	2.94	1.01	0.32	−2.93	8.87
Gender	−10.72	2.95	−3.64	< 0.01	−16.61	−4.82
Agecat	0.68	1.77	0.39	0.70	−2.86	4.22
Disability	−9.02	3.31	−2.73	0.01	−15.64	−2.39
Aidcat	4.71	4.32	1.09	0.28	−3.95	13.37

For TOESRS, gender again showed a significant negative association (*p* = 0.007), with boys scoring lower on environmental supportiveness. This pattern mirrored the gender effect observed in the ToP scores. No significant associations were found for play location, age category, disability status or assistive device use (all *p* > 0.05) (Table 3).

**TABLE 3:** OLS multiple regression results TOES.

Variable	Coefficient	Std. error	*t*	*p*	95% CI lower	95% CI upper
Intercept	12.03	1.23	9.81	< 0.001	9.58	14.49
Location	1.61	1.11	1.45	0.15	−0.61	3.83
Gender	−3.08	1.11	−2.78	0.01	−5.30	−0.86
Agecat	1.17	0.67	1.76	0.08	−0.16	2.51
Disability	−0.13	1.25	−0.11	0.92	−2.63	2.36
Aidcat	0.80	1.63	0.49	0.63	−2.46	4.06

### 3.2 Part II—Qualitative Observation

**FIGURE 7:**
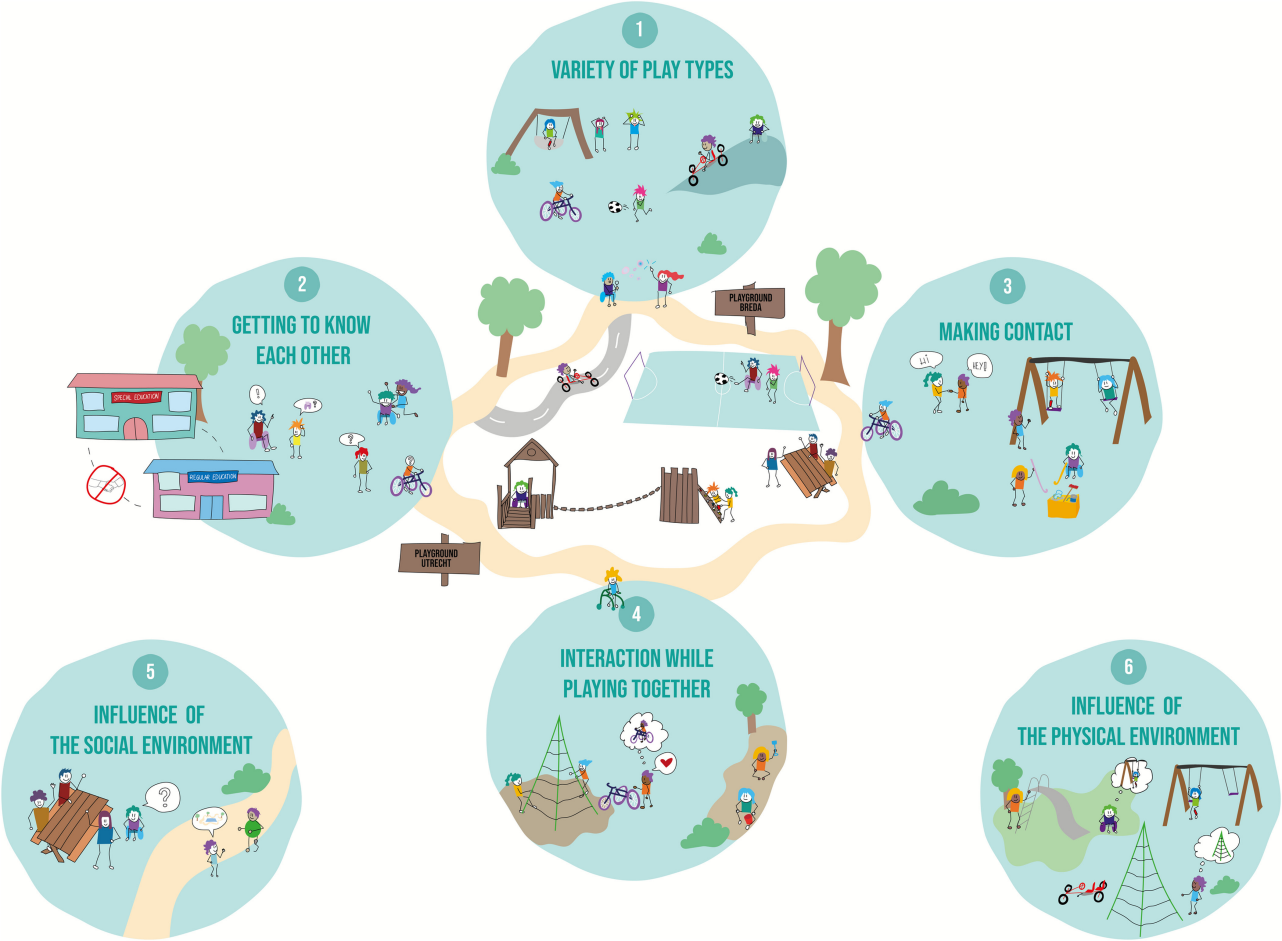
Qualitative observation themes.

The qualitative findings from the data analysis are summarized in Figure 7, which highlights six defined themes: (1) variety of play types, (2) getting to know each other, (3) making contact, (4) interaction while playing together, (5) influence of the social environment and (6) influence of the physical environment. Each theme provides insights into the outdoor play behaviour of children with or without disabilities, as well as the factors that influence their inclusive play. All quotes in this section are drawn from field notes by observers.

1 Variety of play types

Across the sessions, a wide variety of play types was observed, including competitive, fantasy, risky, physically active and less physically active play. In the middle and upper grades, both children with and without disabilities demonstrated competitive behaviours such as attempting to score many soccer goals, pedalling go‐karts at high speed or sliding down hills quickly.

Fantasy play was common in all sessions and often involved imaginative scenarios with vehicles or open spaces: ‘A child with and without disability are still talking. They're discussing dragons and breathing fire, so it seems like a fantasy game. I think they're assigning roles.’

Risky play was prevalent, with children racing go‐karts or wheelchairs, crashing go‐karts and climbing. Children with disabilities attempted the same risky activities as their peers, sometimes taking greater risks, which occasionally resulted in falls. For instance, ‘A child with disability with a scooter, um… tumbles onto the grass, but gets back up. A real go‐getter. It doesn't seem to bother her, yeah, it doesn't seem to matter… she just keeps going.’

To increase the challenge, children sometimes used materials in unconventional ways or combined tasks, such as jumping rope while crossing the wobble bridge or shooting a ball with a go‐kart.

Physically active play, including running, soccer, climbing, swinging, tag and hide‐and‐seek, engaged both children with and without disabilities. In contrast, less active activities like sitting and chatting were primarily observed among children with disabilities. For example, ‘You can actually see that they're trying to push each other on the swing, but now there's also one girl sitting still on the swing, and the girl in the wheelchair and boy with disability are all just standing still.’

2 Getting to know each other

Both children with and without disabilities tended to play with familiar children in small, consistent groups. While some children, regardless of disability status, briefly played apart, they quickly reconnected and showed little interaction with others: ‘They don't really make contact with others, but they're happily playing together.’

At times, children without disabilities interacted with unfamiliar children with disabilities. For example, during an upper‐grade session, a girl with a disability who had been playing alone approached a group of children without disabilities and was welcomed. A conversation followed, during which they collaboratively decided on a game that everyone could participate in and tried it together: ‘A child without disability now asked, “What can you do?” to girl with disability, and now they're going to do something. The child without disability now says, “Okay, and if it's not fun, you should just say so.”’

3 Making contact

Children used both verbal and non‐verbal communication to initiate contact. Children with disabilities more frequently relied on non‐verbal cues, such as eye contact, climbing toward someone or simply observing each other. Verbal communication included chatting, shouting or making jokes, with children using it to introduce themselves, ask questions or request to join a game: ‘A child without disability asks, “Can we join?” A child with disability says it's okay.’

In the lower grade groups, children often initiated contact by asking each other's names or about their schools, with children without disabilities more frequently approaching children with disabilities. In the upper grades, it often involved children without disabilities showing interest in assistive devices of children with disabilities. Choosing the same play materials also led to contact between children with and without disabilities.

Some children hesitated to initiate contact or did not respond to others' attempts to engage. Despite efforts to approach peers, making contact sometimes remained absent, especially among children with disabilities. For example, ‘A child with disability runs toward the castle and tries to engage with the children on the swings but receives no response.’ Similarly, in another play session, a child with disability introduces herself to a child without disability: ‘“My name is… and if you need help, you can ask me,” she says. However, the child without disability runs off.’

4 Interaction while playing together

Interactions varied, ranging from conversations and calling out to discussions and giving instructions. In the middle‐ and upper‐grade groups, conversations often included both children with and without disabilities. Verbal interactions were more common among children without disabilities across all play activities. Beyond verbal communication, children showed consideration by waiting, listening, encouraging, helping, setting boundaries and respecting them. Storytelling also contributed to their play interactions, as children engaged in attentive listening.

When children were engaged in play, it often attracted the attention of other children. After seeing how others played, children were motivated to try it themselves or join in. For example: ‘A child with disability joyfully runs onto the playground and calls out, “I'm back!” … She asks a child without disability if they are going up and says, “Oh, how fun! I'll go up too.”’

Collaboration was particularly evident when children helped peers to join activities. For example, ‘A child with disability is now at the top and gives a child with disability a hand to help climb up. A child without disability is also helping by positioning their feet correctly.’ In addition, children showed interest in the assistive devices of others. For example, they felt the rollator, looked curiously at electric wheelchairs, and interactions occurred through sharing assistive devices: ‘Now, a child without disability is getting on the tricycle of a child with disability. The child with disability has stepped off and says: “It's okay, you can use it!”’.

Some children chose to play alone and did not engage with others. They played side by side happily without seeking interaction. Parallel play occurred most often at the swings in the middle‐ and upper‐grade groups and in the sandbox in the lower‐grade groups. Lower‐grade children and children with disabilities were more likely to observe their social surroundings from a distance, watching other children play. For some children with disabilities, this resulted in not actively participating in the play. For example, one observer mentioned: ‘I also noticed that children often watched each other, and I especially observed that children in wheelchairs tended to watch what the other children were doing.’

Unconstructive play behaviour was also observed. Occasional disagreements, criticism and difficulties connecting occurred between children with and without disabilities. Invitations to play from children with disabilities were more often rejected. Instances of attention‐seeking behaviour were observed among children with disabilities who experienced difficulties joining group play. Some children without disabilities showed a lack of consideration, particularly toward children with disabilities, due to their limited ability to fully engage in activities, such as keeping up with the speed demands of the group: ‘A child without disability and a child with disability are walking next to each other…, running. Another child with disability is trying to keep up but is unable to.’

5 Influence of the social environment

During lower‐grade sessions, children often approached parents to share, seek help or show something, then quickly returned to play. Some children, however, struggled to play without their parents nearby. Several children were initially hesitant to play, requiring guidance from adults: ‘A child without disability finds it a bit scary and goes to his mother to see what she thinks and what he should do. His mother helps him by suggesting what might be fun. He then goes off on his own ….’ Children with disabilities were more likely to ask for help than children without disabilities.

6 Influence of the physical environment

At the start of each session, children enthusiastically ran onto the playground, quickly choosing activities and exploring play materials. Children who were unfamiliar with the schoolyard explored the environment individually and in groups. They showed interest in the new playground and the unfamiliar play equipment, walking or running around as they examined the area to decide where they wanted to play. Familiar children, in contrast, quickly chose their activities.

Certain areas of the playground attracted many children, such as the climbing frames, swings, sandbox, climbing castle and wobbly bridge. Additionally, play materials like go‐karts, bicycles and tricycles were popular. Children with disabilities played more often than children without disabilities with natural materials, such as sticks and flowers, or without specific play materials.

Some children with disabilities encountered physical barriers related to accessibility and playability. For example, they were unable to climb independently, get in and out of the swing and had difficulty getting into a duo pedal cart. However, the children helped each other and tried to make it possible together. For instance, a child with a disability says, ‘I'm not sure… or maybe that can't be done’, to which a child with a disability responds, ‘Come on, I'll help you’. Also, in some situations, adult assistance was required for transfers.

## 4 Discussion

This study examined the inclusive outdoor play behaviour of children with and without disabilities across six play sessions by combining quantitative assessments with qualitative observations. The aim was to explore how children's playfulness and the supportiveness of the play environment were associated with various child and context‐related factors, while also observing inclusivity in play behaviour.

Among all factors, only gender and disability status were significantly associated with ToP scores: Boys and children with disabilities scored lower on playfulness than girls and children without disabilities. This aligns with research suggesting that girls use different social coping strategies than boys, which may positively influence their play (Dean et al. 2017; Saunders et al. 1999), while children with disabilities may face barriers that limit their playfulness (Okimoto et al. 2000). TOES scores were lower for boys but were not significantly associated with disability status or the other variables, indicating they may experience less environmental support, which could affect their play engagement. Play was entirely unstructured, which may have been less aligned with boys' preference for more structured or competitive activities (Boyatzis et al. 1999; Kivikangas et al. 2014). Future research should examine which environmental components best support play for all children, considering individual characteristics such as gender.

While quantitative data highlighted significant differences based on gender and disability status, the observations showed that children engaged in a variety of play types. Children with disabilities were often equally, and sometimes more, willing to take physical risks. They showed motivation to engage in play despite potential limitations. However, moments of less active, solitary and parallel play were more frequently observed among children with disabilities, particularly when physical or social barriers were present. These nuances help explain the lower ToP scores, not as a lack of playfulness per se, but as a reflection of the opportunity to express playfulness in the same ways as children without disabilities. This aligns with TOES findings, where lower scores suggest the environment may not have provided sufficient affordances. Together, ToP and TOES indicate that inclusive play behaviour depends not only on children's playfulness but also on the environmental support they receive, underlining the need to address both aspects when facilitating inclusive outdoor play.

While there were inclusive interactions, peer contact was often limited to familiar playmates for all children, and invitations from children with disabilities were more likely to be ignored or declined. Children without disabilities more frequently initiated verbal contact, while children with disabilities, who were generally able to speak, often used creative non‐verbal cues that were not always noticed by their peers. These subtle social exclusions may have contributed to lower observed playfulness among children with disabilities. Familiarity with the playgrounds and peers could also have influenced interactions: most children with disabilities knew the playgrounds and peers, whereas most children without disabilities were visiting for the first time, and in three cases did not know any of the other participants. Given the limited time and presence of unfamiliar peers, it is unsurprising that children tended to interact with those they already knew (Koster et al. 2010). While this could be considered a limitation, the mix of familiar and unfamiliar peers also reflects natural playground settings, providing insight into real‐world inclusive play dynamics.

Although sessions took place at accessible SPE playgrounds, some children with disabilities could not access certain materials independently, limiting their autonomous play. Peer support and creative problem‐solving helped overcome this. For example, children with and without disabilities assisted each other in moving through the play castle or came up with alternative play activities, illustrating that fostering a culture of inclusivity in play requires not only accessible environments but also supportive social dynamics. Traditionally, therapeutic goals in paediatric rehabilitation focus on function and fitness, such as mobility and strength in PPT, and on functional skills for daily living and participation in OT, while less attention has been given to the social domain of Friends, as highlighted in the F‐words framework (Rosenbaum and Gorter 2012).

A strength of this study is its observational design, which provides detailed information into how children actually play together, that is, their interactions. This approach complements previous interview‐based research by offering an objective perspective on the inclusive play behaviours of Dutch children (Beekhuizen et al. 2025; Engelen et al. 2021; Visser et al. 2025). Additionally, the mixed‐methods approach provided us with a more comprehensive understanding of not only the extent to which children engage in inclusive outdoor play but also to better identify and understand key factors that either facilitate or hinder their participation.

The data were collected at playgrounds of SPE, which may not fully represent play in public playgrounds. While this setting was chosen to eliminate physical accessibility barriers, it may limit generalizability. We also acknowledge possible selection bias, as participating families may already have valued inclusive play. If this motivated group still encountered barriers, these may be even greater among less motivated or resourced families, suggesting our findings represent only the tip of the iceberg.

Future research could focus on conducting interviews with children immediately after engagement in outdoor play, to provide deeper insights into their personal experiences. This approach could reveal children's intentions, motivations and perceived barriers or facilitators that are not always observable, enriching our understanding of how inclusion is experienced from the child's perspective.

## 5 Conclusion

This study provides important insights into the inclusive outdoor play behaviours of children with and without disabilities highlighting the need to understand both social and physical barriers. Quantitative findings revealed lower playfulness scores for boys and children with disabilities, and lower environmental supportiveness scores for boys. Qualitative observations enriched these findings through six key themes: (1) a variety of play types, including fantasy, risky and physically active play; (2) getting to know each other, with unfamiliarity sometimes delaying interaction; (3) making contact, often initiated by children without disabilities with verbal communication, while children with disabilities used creative but sometimes unnoticed non‐verbal cues; (4) interaction while playing together, with more solitary and parallel play when barriers were present; (5) the influence of the social environment, such as peer support and adult presence; and (6) the influence of the physical environment, especially when play structures were not fully accessible. This understanding could be instrumental for paediatric rehabilitation professionals in designing targeted interventions and tools to facilitate more inclusive play opportunities for all children.

## Author Contributions

**Ryan Quint Beekhuizen:** data curation, formal analysis, investigation, methodology, project administration, resources, validation, writing – original draft, writing – review and editing. **Manon Bloemen:** conceptualization, data curation, formal analysis, methodology, project administration, resources, supervision, validation, writing – original draft, writing – review and editing. **Eline Bolster:** conceptualization, investigation, formal analysis, methodology, project administration, resources, supervision, validation, writing – review and editing. **Kirsten Visser:** investigation, formal analysis, methodology, project administration, resources, validation, writing – review and editing. **Remko van der Lugt:** investigation, conceptualization, methodology, writing – review and editing. **Marry Bassa:** investigation, visualization, writing – review and editing. **Nandine Henry:** investigation, resources, writing – review and editing. **Elles Kotte:** formal analysis, writing – review and editing. **Margo van Hartingsveldt:** conceptualization, formal analysis, methodology, validation, writing – review and editing. **Jan Willem Gorter:** conceptualization, formal analysis, methodology, supervision, validation, writing – review and editing.

## Funding

This study was funded by Nationaal Regieorgaan Praktijkgericht Onderzoek SIA (RAAK.PRO04.051).

## Ethics Statement

The Research Ethics Screening Committee in Health at the University of Applied Sciences Utrecht advised that this study was exempt from the Medical Research Involving Human Subjects Act (file number: 221‐000‐2022).

## Conflicts of Interest

The authors declare no conflicts of interest.

## Supporting information


**Appendix S1:** Observation protocol—qualitative observations.

**Data S1:** Supporting information.

**Data S2:** Supporting information.

## Data Availability

The data that support the findings of this study are available on request from the corresponding author. Since these are verbatim transcripts of observations of individuals who may be identifiable, the data are not publicly available due to privacy or ethical considerations.
