# Supplementary material for: Can You See Us Play? Observing Inclusive Outdoor Play Behaviour Among Children With and Without Disabilities: A Mixed Methods Study
Source: Child Care Health Dev. 2025 Dec 4;52(1):e70198. doi: 10.1111/cch.70198 (PMC12676260; doi:10.1111/cch.70198)
Supplement: Supplementary file 1 — Appendix S1: Observation protocol—qualitative observations. [file CCH-52-e70198-s001.docx]

**Observation Protocol – Qualitative Observations**

**Instructions**

- **Describe in detail what you see, using the themes in the observation protocol as a guide.**
- **Keep the description as neutral as possible; avoid adding personal reflections or interpretations.**
- **Use the voice recorder to record your observations: mention the time and location of the observation (e.g., “13:15, near the climbing frame”).**

| **Topic** | **Question** | **Instruction** |
| --- | --- | --- |
| **Play Together** | | |
| **Initiating contact** | How do children approach each other?  Who takes the initiative?  Do children share something about themselves (e.g., their disability)? | *Describe how children seek contact and how play is initiated.* |
| **Play between children with/without disabilities** | How do children play together? Or do they play separately? What types of play do they show? | *Describe the ways in which children play together. For example: in pairs, in groups, changing constellations. How long does the joint play last? How does it start and how does it end?*  *[Optional] Describe type of play: solitary, onlooker, parallel, group play (associative, cooperative).* |
| **Type of activities/play** | What kinds of activities do the children engage in together? | *Beschrijf het type activiteit in detail: bijv. “kind A en kind B Describe the activity in detail. For example: “Child A and Child B play together pretending to be knights, using sticks as swords.”*  *[Optional] Describe type of play: pretend play, physical play, constructive play, competitive play, symbolic play.* |
| **Social Interactions** | | |
| **Interaction style** | How do the children interact with each other? What goes well? What does not? | *Describe social interactions, e.g., having fun together, conflict, bullying.* |
| **Considering one another** | Do children take each other into account? Do they help each other? | *Describe how children consider each other (and each other’s disabilities). For example, helping pick up a ball, adjusting the game.* |
| **Emotions** | What emotions are visible in (joint) play? | *Describe the emotions observed. For example: joy, frustration (e.g., due to not being able to do something), fear/hesitation.* |
| **Physical Environment** | | |
| **Use of materials/equipment** | How do children use materials/equipment? | *Describe how children use materials and/or playground equipment. Does this stimulate (joint) play? Are there things children with disabilities cannot do? How is this handled?* |
| **Use of the playground space** | How do children use the available outdoor space/school playground? | *Describe how children use the playground. Which areas do they visit? Which do they avoid? Is the entire area used?* |
| **Physical intensity** | What is the physical intensity of the play? What provides challenge? | *Describe and estimate physical intensity:*   - ***Low:*** *sitting, low-movement activities* - ***Moderate:*** *walking, typical wheelchair movement* - ***High:*** *running, sports, fast wheelchair movement*   *Also note signs such as increased breathing rate, flushed cheeks, sweating. Describe what stimulates physically intensive play (specific games, materials).* |
| **Independent Play** | | |
| **Independent play for children with disabilities** | To what extent can children with disabilities play independently? | *Describe the degree to which children with disabilities can play independently. What can they do themselves, and where do they need support from adults or peers?* |
| **Adult intervention** | Is adult help or intervention needed? | *Describe whether adult intervention is necessary and in which situations this occurs.* |
